# Supplementary material for: Reverse Sap Flow from Fruit
Source: Plants (Basel). 2025 Dec 30;15(1):105. doi: 10.3390/plants15010105 (PMC12787672; doi:10.3390/plants15010105)
Supplement: Supplementary file 1 [file plants-15-00105-s001.zip › plants-4029223-supplementary.pdf]

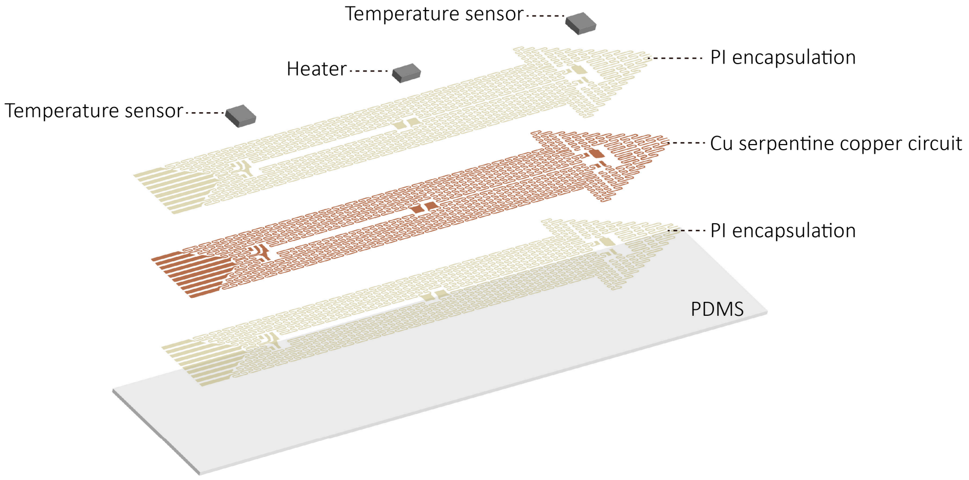

Supplementary Figure S1. Exploded view illustration of the sap flow sensor. The sensor is composed of three distinct layers: (1) a thick, stretchable base layer made of polydimethylsiloxane (PDMS) with a thickness of 10 µm, which provides mechanical support and ensures reversible adhesion to the plant surface; (2) a laser-etched serpentine copper circuit, 6 µm in thickness, sandwiched between two polyimide (PI) layers, each approximately 1 µm thick, forming a stretchable conductive pathway for electrical communication; and (3) a top layer that integrates two micro-temperature sensors along with a heater, designed for measuring sap flow.

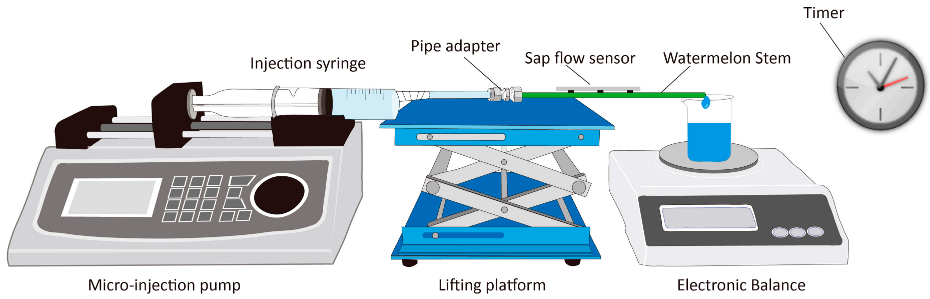

Supplementary Figure S2. Schematic diagram of the experimental setup for sap flow sensor calibration. In the experiment, a freshly harvested watermelon stem, measuring 10 cm in length and about 5 mm in diameter, was connected to a syringe filled with water through a pipe adapter. The syringe's injection rate was controlled using a micro-injection pump (LSP01-1B, China), and different water injection rates were set to simulate sap flow through the stem. A sap flow sensor was installed at the middle section of the stem for measurement. The calibration procedure was as follows: First, the injection rate of the micro-injection pump was set from low to high, and the actual sap flow rate was calculated by measuring the total mass of water collected by a balance within a unit time. Second, at each sap flow rate, the sensor was used to repeatedly measure multiple sets of time-temperature difference ($\Delta T = T_{downstream} - T_{upstream}$) curves, from which the ascending slope of each curve was derived, and the mean and standard deviation were obtained. Third, the sap flow rates and the corresponding mean ascending slopes of the ΔT curves were plotted as scatter points (Figure 2E), and a linear regression was performed to obtain the calibration curve and the sap flow calculation equation. Finally, by comparing the sensor-predicted values with the known flow rates, the mean relative error (MRE) of the sensor measurements was 0.12, and the limit of detection (LOD) was calculated as 5 μL/min based on the 3-sigma rule. The primary source of measurement error in this sensor arises from whether the three components—the two temperature sensors and the heater—maintain tight contact with the stem surface during installation.

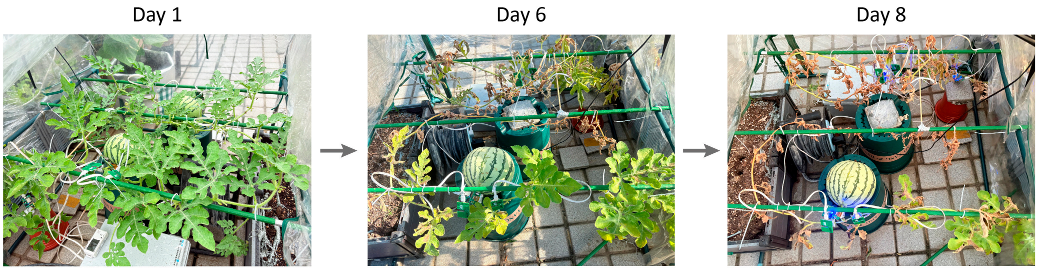

Supplementary Figure S3. Photographs showing the growth conditions of fruit-bearing and defruited plants subjected to extended drought stress. By Day 6, the majority of the leaves near the apex of the defruited plant had wilted, while only a few leaves of the fruit-bearing plant showed signs of wilting. By Day 8, as drought conditions intensified, the defruited plant had died, while the plant with fruit retained live leaves from the roots to the fruit region.
